# Curcumin and its combination with a reduced dose of rosuvastatin: A promising therapy for chronic kidney disease and associated dyslipidemia in rat animal models

**DOI:** 10.17305/bb.2024.11091

**Published:** 2024-10-01

**Authors:** Dina Lagumdžija, Aida Hamzić Mehmedbašić, Džan Ahmed Jesenković, Bakir Kudić, Dina Kapić, Esad Ćosović, Orhan Lepara, Belma Pehlivanović Kelle, Jasminka Prguda-Mujić, Jasna Kusturica, Naida Herenda Pušina, Fahir Bečić, Aida Kulo Ćesić

**Affiliations:** 1 Department of Pharmacology and Clinical Pharmacy, University of Sarajevo, Faculty of Pharmacy, Sarajevo, Bosnia and Herzegovina; 2 Clinic of Nephrology, Clinical Center University of Sarajevo, Sarajevo, Bosnia and Herzegovina; 3 Department for Epidemiology and Biostatistics, University of Sarajevo, Faculty of Medicine, Sarajevo, Bosnia and Herzegovina; 4 Department of Pharmacology and Toxicology, University of Sarajevo, Faculty of Medicine, Sarajevo, Bosnia and Herzegovina; 5 Department of Histology and Embryology, University of Sarajevo, Faculty of Medicine, Sarajevo, Bosnia and Herzegovina; 6 Department of Physiology, University of Sarajevo, Faculty of Medicine, Sarajevo, Bosnia and Herzegovina; 7 Institute for Biomedical Diagnostic and Research Medicover BH, Sarajevo, Bosnia and Herzegovina; 8 Clinic for Anesthesiology and Reanimatology, Clinical Center University of Sarajevo, Sarajevo, Bosnia and Herzegovina

**Keywords:** Chronic kidney disease (CKD), adenine, curcumin, rosuvastatin, dyslipidemia

## Abstract

The objective of this study was to confirm the effects of curcumin and to investigate the effects of its combination with a reduced dose (RD) of rosuvastatin in an adenine-induced model of chronic kidney disease (CKD) and associated dyslipidemia in rats. Renal function and morphology, as well as lipid status, were assessed using laboratory parameters and histopathological analysis. Male Wistar rats (*n* ═ 36) randomly divided into six groups, were treated for 24 days: normal control (standard diet), CKD control (adenine diet, 0.75% w/w adenine-supplemented diet), curcumin (100 mg/kg/day + adenine diet), rosuvastatin minimal therapeutic dose (MTD) (5 mg/day + adenine diet), rosuvastatin RD (25% of rosuvastatin MTD + adenine diet), and rosuvastatin RD + curcumin (25% of rosuvastatin MTD + curcumin 100 mg/kg/day + adenine diet) group. While rosuvastatin alone showed only antilipemic action, both curcumin alone and its combination with an RD of rosuvastatin showed better renal protection with lower serum creatinine levels and milder renal morphological alterations, as well as better antilipemic action with lower levels of triglycerides, very low-density lipoprotein, and low-density lipoprotein cholesterols compared with the levels in CKD control rats. Treatment with curcumin alone also resulted in a significantly higher estimated glomerular filtration rate, lower uric acid levels, and higher high-density lipoprotein cholesterol, while the combined therapy additionally resulted in higher serum albumin levels, lower total cholesterol, and both atherogenic and coronary risk indexes compared with CKD control rats. The results of this study confirmed the beneficial effects of curcumin alone and provided new evidence for the beneficial effects of its combination with an RD of rosuvastatin in rats with CKD and associated dyslipidemia.

## Introduction

With approximately 850 million people affected by chronic kidney disease (CKD), it is a major public health issue with a globally increasing prevalence and high mortality rate [1]. Its multiple adverse outcomes, such as progressive and irreversible loss of kidney function and cardiovascular complications lead to premature death [2]. To date, there are no available therapies for improving kidney function in patients with CKD, and the current therapeutic options are limited to the management of dyslipidemia, hypertension, diabetes mellitus, and other CKD complications [3, 4]. Prevention and treatment of CKD complications are of great relevance, as these patients have a higher risk of developing cardiovascular disease than reaching end-stage kidney disease, and most patients with CKD die from cardiovascular complications [5]. Therefore, to reduce cardiovascular risk, these patients are frequently treated with statins, most often rosuvastatin, the most potent of the currently available statins, despite studies showing its numerous side effects, including deterioration of kidney function, commonly presented in the form of proteinuria [6], and kidney morphology changes [7, 8]. Curcuma longa, also known as Turmeric from the Zingiberaceae (ginger) family, is a widely used spice in traditional medicine for the treatment of many diseases, such as ulcers, various tumors, bacterial and viral infections, diabetes, and arthritis [9]. Curcumin (diferuloyl methane), its main curcuminoid, has been shown to possess antioxidant, anti-inflammatory [10], antimicrobial, antimutagenic, anticarcinogenic, hypolipidemic, and antifibrotic properties [11]. Curcumin prevents liver damage, promotes muscle regeneration, and has beneficial effects on multiple sclerosis, Alzheimer’s disease, inflammatory bowel disease, lung fibrosis, and psoriasis. Results of *in vitro* and *in vivo* studies also suggest its beneficial effects on many types of cancer, including kidney cancer, leukemia, prostate cancer, melanoma, breast, liver, and colon cancer [12]. Multiple animal studies, including those with adenine-induced CKD [13, 14], have demonstrated its ability to improve renal function and reduce CKD-associated cardiovascular complications, including dyslipidemia [14, 15]. In 5/6 nephrectomized rats, curcumin reduces proteinuria, serum creatinine, and blood urea nitrogen (BUN) levels, and decreases renal apoptosis and fibrosis [16, 17]. Furthermore, it has shown renoprotective effects in other rat models, including doxorubicin-induced kidney injury [18], cyclosporine A-induced nephrotoxicity [19], and cisplatin-induced nephrotoxicity in mice [20].

Based on a drug–drug interaction study in rats and dogs, where co-administration of curcumin significantly increased rosuvastatin plasma concentration, area under the curve, and serum half-life [21], and given that the earlier studies showed its conflicting renal safety profile, we hypothesized that their combination could give the possibility of rosuvastatin dose reduction and, consequently, a reduction in its renal side effects. Furthermore, no studies have investigated the use of a combination of rosuvastatin and curcumin in CKD and associated dyslipidemia.

Therefore, the objective of this study was to confirm the effects of curcumin and to investigate the effects of its combination with a reduced dose (RD) of rosuvastatin in an adenine-induced model of CKD and associated dyslipidemia in rats.

## Materials and methods

### Chemicals

Adenine (CAS No. 73-24-5), curcumin (CAS No. 458-37-7), rosuvastatin (CAS No. 147098-20-2), and dimethyl sulfoxide (DMSO) (CAS No. 67-68-5) were purchased from Sigma-Aldrich (St. Louis, MO, USA). Biochemical assay kits for determining levels of serum urea, creatinine, albumin, uric acid, total cholesterol, and triglycerides were purchased from Sigma-Aldrich, and a diagnostic kit for high-density lipoprotein (HDL) determination was purchased from Abcam, England. The standard rat diet was obtained from Versele Laga Nature and contained approximately 60% carbohydrates, 17.5% protein, 8.5% fat, and 7% crude fiber (by weight percentage). The adenine-supplemented diet contained 0.75% w/w adenine in the standard diet. All other chemicals were of the highest purity grade available.

### Animals

Thirty-six male Wistar rats, three months old, were obtained from the University of Sarajevo–Faculty of Medicine to model adenine-induced CKD. The animals were housed in a temperature-controlled room (22 ± 1 ^∘^C) with a 12-h light/dark cycle. Prior to the experiment, an acclimatization period was conducted for seven days. Animals were fed standard rat chow ad libitum and had unlimited access to water.

### Adenine-induced CKD model and study design

Rats were randomly divided into six groups (one normal control group and five CKD-induced groups) of six animals each. Group I (normal control) received DMSO along with standard rodent food. All CKD-induced groups (groups II–VI) received an adenine-supplemented diet (0.75% w/w adenine in the standard diet) in order to induce CKD [22]. In addition to the adenine-supplemented diet, group II received DMSO and served as the CKD control. Group III received curcumin at a dose of 100 mg/kg/day, group IV received the minimal therapeutic dose (MTD) of rosuvastatin at 5 mg/day, group V received the reduced MTD (RD, 25% of MTD, i.e., 1.25 mg/day) of rosuvastatin, and group VI received a combination of rosuvastatin RD (1.25 mg/day) and curcumin (100 mg/kg/day), all dissolved in DMSO. The curcumin dose (100 mg/kg/day) was chosen based on previously reported data regarding its positive effects on dyslipidemia in rats with CKD [14], and the human doses of rosuvastatin MTD and RD were converted to rat doses using the factor of conversion 6.2 as previously reported [23]. All treatments were administered orally via a sterile plastic tube (Intech Laboratories, Inc., USA) once daily for 24 days. On day 22, rats were placed individually in metabolic cages for an acclimatization period of two days, and on the 25th day, 24-h urine samples were collected. Urine samples were stored at -20 ^∘^C until analysis. The rats were anesthetized with a cocktail of ketamine (75 mg/kg) and xylazine (5 mg/kg) injected intraperitoneally, and blood samples (approximately 4 mL) were collected via cardiac puncture. Blood was allowed to clot at room temperature before being centrifuged at 4000 rpm for 10 min. Serum was separated and stored at -20 ^∘^C for biochemical analysis. After blood collection, the kidney of each rat was dissected, weighed, and stored in formalin until histopathological analysis.

### Measurement of body mass, water, and food intake

The body mass of all rats was recorded daily before treatment at 08:00 A.M. for 25 days.

The 24-h water and food intake were recorded daily at 10:00 A.M.

### Biochemical analysis

BUN, serum creatinine, serum uric acid, serum albumin, urine albumin, total serum cholesterol, serum triglycerides, and serum HDL were measured using commercial diagnostic kits.

Estimated glomerular filtration rate (eGFR), as a clinical index of kidney function, was calculated using the rats’ body mass, serum creatinine, and BUN values according to the following formula: Serum creatinine < 52 µmol/L: eGFR ═ 880 × M^0.695^ × C^-0.660^ × U^-0.391^

Serum creatinine ≥ 52 µmol/L: eGFR ═ 5862 × M^0.695^ × C^-1.150^ × U^-0.391^ where eGFR is the estimated GFR (µL/min), M is body mass (g), C is creatinine concentration (µmol/L), and U is BUN concentration (mmol/L) [24].

Serum very low-density lipoprotein (VLDL) cholesterol and low-density lipoprotein (LDL) cholesterol were calculated indirectly using Friedewald’s equations. VLDL ═ Triglycerides/5; LDL ═ Total cholesterol – [HDL + VLDL]. The atherogenic index (AI) and coronary risk index (CRI), as measures of the extent of atherosclerotic lesions and coronary atherosclerosis development, respectively, were calculated using serum total cholesterol and HDL cholesterol from different groups of rats, using the following formula: AI ═ [Total cholesterol – HDL]/HDL; CRI ═ Total cholesterol/HDL [25].

### Kidney mass and histopathology

The removed kidneys were bisected and the weight of each kidney was recorded. The kidneys were then fixed in 10% formaldehyde at room temperature before being dehydrated and embedded in paraffin. Paraffin blocks were made and cut into 5-µm-thick sections. The sections were stained with hematoxylin/eosin, Azan, and periodic acid-Schiff (PAS) and examined under a light microscope. Qualitative and semiquantitative histological analyses were performed in a blinded manner. The following histologic parameters were observed and analyzed: inflammation, interstitial fibrosis, tubular degeneration, tubular dilation, and the presence of crystals. The semiquantitative histological scoring was performed according to the following criteria: 0 ═ no changes, 1 ═ minimal changes with one or a few small foci, 2 ═ mild changes with small to moderately sized foci, 3 ═ moderate changes with frequent and moderately sized foci, and 4 ═ severe changes with extensive confluent foci affecting most of the kidney, modified from Blasi et al. [26].

### Ethical statement

The experimental procedure was approved by the University of Sarajevo–Medical Faculty Ethics Committee (Approval Number: 02-3-4-3304/22), following the guidelines of The Council for International Organizations of Medical Sciences (CIOMS) and The International Council for Laboratory Animal Science (ICLAS); International Guiding Principles for Biomedical Research Involving Animals.

### Statistical analysis

Statistical analysis was conducted in R 4.4.0 (R Foundation for Statistical Computing, Vienna, Austria). Descriptive summaries were reported as mean and standard deviation (SD), or median and interquartile range (IQR, Q1–Q3) for numerical variables, as applicable. The significance threshold was set at α ═ 0.05, with lower *P* values deemed as statistically significant at this level.

Quantitative variables were assessed for normality using histograms, QQ plots, and Shapiro–Wilk’s test, while homogeneity of variances was evaluated using Levene’s test prior to employing bivariate tests for differences in means between groups. Differences in means were tested using Fisher’s one-way ANOVA test for variables with equal variances, and Welch’s one-way ANOVA test for variables with unequal variances. Variables that violated the assumptions for parametric tests were tested with the Kruskal–Wallis test.

Tests for differences in means were followed up with appropriate post-hoc tests, adjusted for false discovery rate (FDR) for type I error. FDR for type I error inflation was controlled using Benjamini–Hochberg criteria. Dunn’s test was used as a post-hoc test to investigate pairwise differences between groups following a significant Kruskal–Wallis test.

## Results

Our study included 36 Wistar rats, out of which 4 died during the study. Attrition was recorded in the CKD control, curcumin, rosuvastatin RD, and rosuvastatin RD + curcumin treatment groups, each containing five rats at the end of the study, while the normal control and rosuvastatin RD groups completed the experiment without attrition.

### Body mass

**Figure 1.**
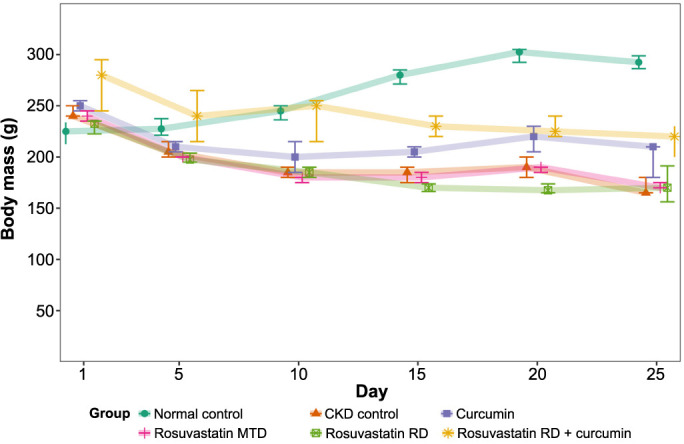
**The effects of curcumin, rosuvastatin in an MTD, rosuvastatin in an RD, and the combination of rosuvastatin RD and curcumin on the body mass of rats with adenine-induced CKD during the experiment.** The normal control group was fed a standard diet, and the CKD control group was fed 0.75% w/w adenine in the standard diet. MTD: Minimal therapeutic dose; RD: Reduced dose; CKD: Chronic kidney disease.

All treatment groups, except the normal control, lost body mass during the experiment. At the start of the experiment, the highest median body mass was recorded in the rosuvastatin RD + curcumin group (280.0, IQR 245.0–295.0 g), while the lowest (225.0, IQR 212.5–233.8 g) was recorded in the normal control group. Differences at all timepoints were significant. Summarized values across days are displayed in Figure 1. At the end of the experiment, the highest median body mass was recorded in the normal control group (292.5, IQR 286.3–298.8 g), while the lowest (165.0, IQR 165.0–180.0 g) was recorded in the CKD control group. Post-hoc statistical testing showed significant differences between the normal control group on one side and the CKD control group and groups treated with rosuvastatin MTD and rosuvastatin RD on the other side (292.5, IQR 286.3–298.8 vs 165, IQR 165–180; 170, IQR 170–175; 170 IQR 156.3–191.3; *P* ═ 0.004 for all). No significant differences were found between other groups on the last day of the experiment (Figure 1).

### Water and food intake

**Figure 2.**
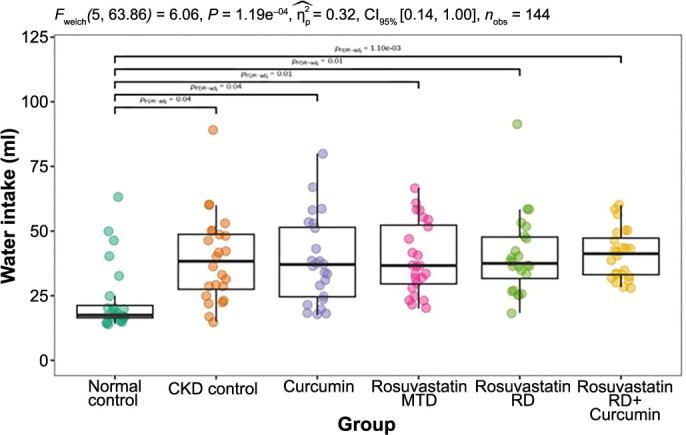
**The effects of curcumin, rosuvastatin in an MTD, rosuvastatin in an RD, and the combination of rosuvastatin RD and curcumin on the group mean daily water intake (mL) in rats with adenine-induced CKD, over the duration of the experiment (24 days).** The normal control group was fed a standard diet, and the CKD control group was fed 0.75% w/w adenine in the standard diet. MTD: Minimal therapeutic dose; RD: Reduced dose; CKD: Chronic kidney disease.

Mean daily water intake was comparable across all CKD-induced groups, starting from the lowest: 38.5 (±17) mL in the curcumin group, 39.2 (±17) mL in the CKD control group, 39.4 (±14) mL in the rosuvastatin MTD group, and 40.5 (±15) mL in the rosuvastatin RD group. The highest mean daily water intake was observed in the rosuvastatin RD + curcumin group 41 (±10) mL. In contrast, the normal control group had a lower mean daily water intake of 23.5 (±13) mL (Figure 2).

**Figure 3.**
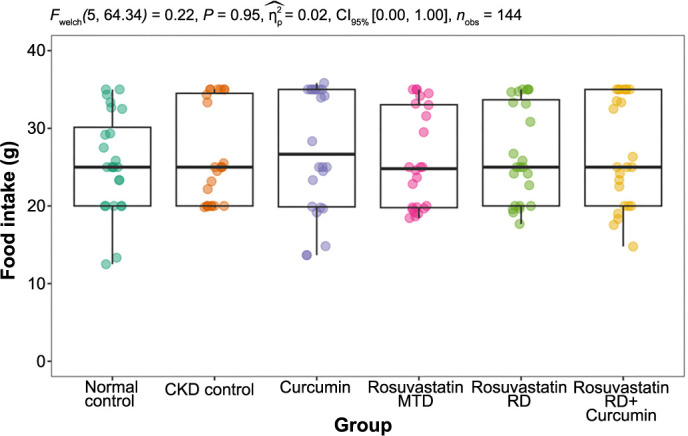
**The effects of curcumin, rosuvastatin in an MTD, rosuvastatin in an RD, and the combination of rosuvastatin RD and curcumin on the group mean daily food intake (g) in rats with adenine-induced CKD, over the duration of the experiment (24 days).** The normal control group was fed a standard diet, and the CKD control group was fed 0.75% w/w adenine in the standard diet. MTD: Minimal therapeutic dose; RD: Reduced dose; CKD: Chronic kidney disease.

Mean daily food intake was similar among groups. It was the lowest in the normal control group 25.5 (±6.4) g while in CKD-induced groups, the lowest mean daily food intake was found in the rosuvastatin MTD group 25.9 (±6.4) g, followed by the CKD control 26.4 (±6.3) g, rosuvastatin RD 26.7 (±6.3) g, rosuvastatin RD + curcumin 26.9 (±7.0) g, and finally, the highest value was found in the curcumin group 27.3 (±7.9) g (Figure 3).

### Biochemical measurements and eGRF

The group fed an adenine-supplemented diet (CKD control) had significantly higher concentrations of BUN (23, IQR 18–31 vs 4, IQR 3–6; *P* < 0.001), serum creatinine (84, IQR 75–87 vs 33, IQR 20–42; *P* ═ 0.01), serum uric acid (149, IQR 148–155 vs 89, IQR 78–105; *P* ═ 0.037), and urine albumin (12.8, IQR 9.4–14.2 vs 4.1, IQR 3.5–4.5; *P* ═ 0.005), and significantly lower eGFR (24, IQR 19–31 vs 213, IQR 126–297; *P* ═ 0.002) compared with the normal control group. No significant difference in the concentration of serum albumin was seen between the CKD control group and the normal control group (0.80, IQR 0.80–0.80 vs 3.55, IQR 3.23–4.03; *P* ═ 0.145).

The group treated with curcumin had significantly lower concentrations of serum creatinine (30, IQR 23–59 vs 84, IQR 75–87; *P* ═ 0.042) and serum uric acid (86, IQR 68–102 vs 149, IQR 148–155; *P* ═ 0.047), and significantly higher eGFR (122, IQR 57–126 vs 24, IQR 19–31; *P* ═ 0.038) compared to the CKD control group. No significant difference in the concentrations of BUN (10, IQR 7–11 vs 23, IQR 18–31; *P* ═ 0.067), serum albumin (2.90, IQR 2.80–4.80 vs 0.80, IQR 0.80–0.80; *P* ═ 0.145), and urine albumin (5.5, IQR 5.2–5.6 vs 12.8, IQR 9.4–14.2; *P* ═ 0.072) was seen between the curcumin group and the CKD control group.

The group treated with rosuvastatin MTD had no significant difference in the concentrations of BUN (12, IQR 11–12 vs 23, IQR 18–31; *P* ═ 0.148), serum creatinine (75, IQR 73–78 vs 84, IQR 75–87; *P* ═ 0.72), urine albumin (12.3, IQR 7.8–15.1 vs 12.8, IQR 9.4–14.2; *P* ═ 0.906), and eGFR (37, IQR 35–39 vs 24, IQR 19–31; *P* ═ 0.483) compared to the CKD control group. A significantly higher concentration of serum albumin (5.50, IQR 3.20–5.60 vs 0.80, IQR 0.80–0.80; *P* ═ 0.018) was seen in the group treated with rosuvastatin MTD compared to the CKD control group.

The group treated with rosuvastatin RD had no significant difference in the concentrations of BUN (28, IQR 22–32 vs 23, IQR 18–31; *P* ═ 0.8), serum creatinine (80, IQR 71–85 vs 84, IQR 75–87; *P* ═ 0.97), urine albumin (11.1, IQR 10.4–11.8 vs 12.8, IQR 9.4–14.2; *P* ═ 0.819), and eGFR (24, IQR 22–33 vs 24, IQR 19–31; *P* ═ 0.939). A significantly higher concentration of serum albumin (4.70, IQR 3.50–6.35 vs 0.80, IQR 0.80–0.80; *P* ═ 0.006) was seen in the group treated with rosuvastatin RD compared to the CKD control group.

**Figure 4.**
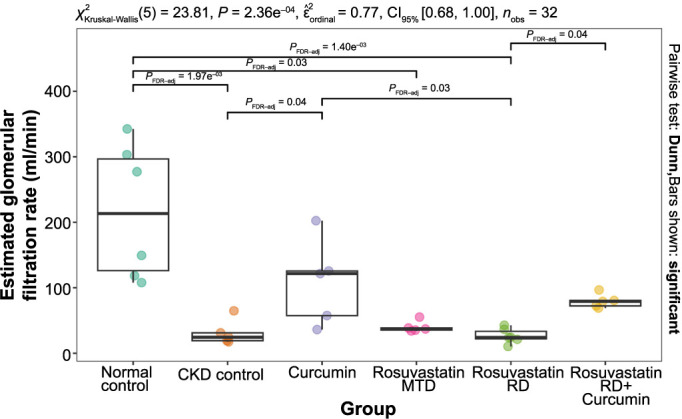
**The effects of curcumin, rosuvastatin in an MTD, rosuvastatin in an RD, and the combination of rosuvastatin in RD and curcumin on the eGFR (mL/min) in rats with adenine-induced CKD.** The normal control group was fed a standard diet, and the CKD control group was fed 0.75% w/w adenine in the standard diet. MTD: Minimal therapeutic dose; RD: Reduced dose; CKD: Chronic kidney disease.

The group treated with a combination of rosuvastatin RD and curcumin had a significantly lower concentration of serum creatinine (48, IQR 45–49 vs 84, IQR 75–87; *P* ═ 0.043) and a significantly higher concentration of serum albumin (5.30, IQR 5.10–5.40 vs 0.80, IQR 0.80–0.80; *P* ═ 0.006) compared to the CKD control group. No significant difference in the concentrations of BUN (10, IQR 8–12 vs 3, IQR 18–31; *P* ═ 0.067), urine albumin (5.2, IQR 4.5–9.3 vs 12.8, IQR 9.4–14.2; *P* ═ 0.074), and eGFR (79, IQR 72–80 vs 24, IQR 19–31; *P* ═ 0.051) was seen between group treated with the combination of rosuvastatin RD and curcumin and the CKD control group (Table 1 and Figure 4).

**Table 1:** The effects of curcumin, rosuvastatin in an MTD, rosuvastatin in an RD, and the combination of rosuvastatin RD and curcumin on serum and urine biomarkers of renal function in rats with adenine-induced CKD. The normal control group was fed a standard diet, and the CKD control group was fed 0.75% w/w adenine in the standard diet

**Treatment group**							
**Variable**	**Normal control (*N* ═ 6)**	**CKD control (*N* ═ 5)**	**Curcumin (*N* ═ 5)**	**Rosuvastatin MTD (*N* ═ 5)**	**Rosuvastatin RD (*N* ═ 6)**	**Rosuvastatin RD + Curcumin (*N* ═ 5)**	***P* value^1^**
Blood urea nitrogen (mmol/L)	4 (3–6)	23^###^ (18–31)	10^ns^ (7–11)	12^ns^ (11–12)	28^ns^ (22–32)	10^ns^ (8–12)	<0.001
Serum creatinine (µmol/L)	33 (20–42)	84^##^ (75–87)	30^*^ (23–59)	75^ns^ (73–78)	80^ns^ (71–85)	48^*^ (45–49)	<0.001
Serum uric acid (µmol/L)	89 (78–105)	149^#^ (148–155)	86^*^ (68–102)	133^ns^ (130–137)	123^ns^ (110–131)	115^ns^ (105–143)	<0.017
Serum albumin (g/dL)	3.55 (3.23–4.03)	0.80^ns^ (0.80–0.80)	2.90^ns^ (2.80–4.80)	5.50^*^ (3.20–5.60)	4.70^**^ (3.50–6.35)	5.30^**^ (5.10–5.40)	<0.005
Urine albumin (ng/mL)	4.1 (3.5–4.5)	12.8^##^ (9.4–14.2)	5.5^ns^ (5.2–5.6)	12.3^ns^ (7.8–15.1)	11.1^ns^ (10.4–11.8)	5.2^ns^ (4.5–9.3)	<0.001

Data represent median (Q1–Q3) of 5–6 rats; ^1^Kruskal–Wallis rank sum test; Significant difference from normal control: ^#^*P* < 0.05; ^##^*P* < 0.01; ^###^*P* < 0.001; Significant difference from CKD control: *P < 0.05; **P < 0.01; ***P < 0.001; No significant difference from CKD control: ns (*P* > 0.05). MTD: Minimal therapeutic dose; RD: Reduced dose; CKD: Chronic kidney disease.

### Lipid status

The group fed an adenine-supplemented diet (CKD control) had significantly lower serum HDL cholesterol (0.12, IQR 0.11–0.12 vs 0.67, IQR 0.58–0.72; *P* < 0.001) and significantly higher serum LDL cholesterol (1.02, IQR 0.85–1.03 vs 0.35, IQR 0.34–0.36; *P* ═ 0.047), AI (11.8, IQR 11.5–12.5 vs 0.5, IQR 0.5–0.6; *P* ═ 0.008), and CRI (7.92, IQR 7.76–8.53 vs 0.56, IQR 0.51–0.59; *P* ═ 0.007) compared to the normal control group.

The group treated with curcumin had significantly higher levels of serum HDL cholesterol (0.45, IQR 0.43–0.51 vs 0.12, IQR 0.11–0.12; *P* ═ 0.045) and lower levels of serum triglycerides (0.37, IQR 0.32–0.43 vs0.89, IQR 0.86–1.21; *P* ═ 0.047), serum VLDL cholesterol (0.07, IQR 0.06–0.09 vs 0.18, IQR 0.17–0.24; *P* ═ 0.047), and serum LDL cholesterol (0.35, IQR 0.33–0.36 vs 1.02, IQR 0.85–1.03; *P* ═ 0.045) compared to the CKD control group. However, there was no significant difference in levels of total serum cholesterol (0.81, IQR 0.78–0.84 vs 1.54, IQR 1.48–1.59; *P* ═ 0.114), and both AI (0.9, IQR 0.6–0.9 vs 11.8, IQR 11.5–12.5; *P* ═ 0.065) and CRI (0.73, IQR 0.64–0.82 vs 7.92, IQR 7.76–8.53; *P* ═ 0.085).

The group treated with rosuvastatin MTD had significantly better lipid profiles with lower levels of total serum cholesterol (0.49, IQR 0.43–0.58 vs 1.54, IQR 1.48–1.59; *P* ═ 0.004), serum triglycerides (0.35, IQR 0.34–0.42 vs 0.89, IQR 0.86–1.21; *P* ═ 0.024), serum VLDL cholesterol (0.07, IQR 0.07–0.08 vs 0.18, IQR 0.17–0.24; *P* ═ 0.024), serum LDL cholesterol (0.10, IQR 0.09–0.14 vs 1.02, IQR 0.85–1.03; *P* ═ <0.001), and both AI (0.1, IQR 0.1–0.1 vs 11.8, IQR 11.5–12.5; *P* ═ <0.001) and CRI (0.23, IQR 0.22–0.28 vs 7.92, IQR 7.76–8.53; *P* ═ <0.001), while levels of serum HDL cholesterol were higher (0.46, IQR 0.40–0.52 vs 0.12, IQR 0.11–0.12; *P* ═ 0.045) compared to the CKD control group.

No significant difference in the lipid status of total serum cholesterol (0.88, IQR 0.80–0.94 vs 1.54, IQR 1.48–1.59; *P* ═ 0.12), serum triglycerides (0.64, IQR 0.42–1.05 vs 0.89, IQR 0.86–1.21; *P* ═ 0.197), serum HDL cholesterol (0.41, IQR 0.37–0.46 vs 0.12, IQR 0.11–0.12; *P* ═ 0.161), serum VLDL cholesterol (0.13, IQR 0.08–0.21 vs 0.18, IQR 0.17–0.24; *P* ═ 0.197), serum LDL cholesterol (0.41, IQR 0.35–0.43 vs 1.02, IQR 0.85–1.03; *P* ═ 0.113) or AI (1.2, IQR 0.8–1.5 vs 11.8, IQR 11.5–12.5; *P* ═ 0.255) and CRI (0.90, IQR 0.66–1.28 vs 7.92, IQR 7.76–8.53; *P* ═ 0.18) was found between the rosuvastatin RD group and the CKD control group.

The group treated with a combination of rosuvastatin RD and curcumin had significantly lower levels of total serum cholesterol (0.75, IQR 0.69–0.84 vs 1.54, IQR 1.48–1.59; *P* ═ 0.018), serum triglycerides (0.30, IQR 0.27–0.32 vs 0.89, IQR 0.86–1.21; *P* ═ 0.009), serum VLDL cholesterol (0.06, IQR 0.05–0.06 vs 0.18, IQR 0.17–0.24; *P* ═ 0.009), serum LDL cholesterol (0.26, IQR 0.24–0.40 vs 1.02, IQR 0.85–1.03; *P* ═ 0.025), and both AI (0.5, IQR 0.4–1.0 vs 11.8, IQR 11.5–12.5; *P* ═ 0.032) and CRI (0.58, IQR 0.49–0.97 vs 7.92, IQR 7.76–8.53; *P* ═ 0.038) compared with the CKD control group (Table 2).

**Table 2:** The effects of curcumin, rosuvastatin in an MTD, rosuvastatin in an RD, and the combination of rosuvastatin RD and curcumin on the lipid profile in rats with adenine-induced CKD. The normal control group was fed a standard diet, and the CKD control group was fed 0.75% w/w adenine in the standard diet

**Treatment group**							
**Variable**	**Normal control (*N* ═ 6)**	**CKD control (*N* ═ 5)**	**Curcumin (*N* ═ 5)**	**Rosuvastatin MTD (*N* ═ 5)**	**Rosuvastatin RD (*N* ═ 6)**	**Rosuvastatin RD + Curcumin (*N* ═ 5)**	***P* value^1^**
Total serum cholesterol (mmol/L)	0.98 (0.91–1.05)	1.54^ns^ (1.48–1.59)	0.81^ns^ (0.78–0.84)	0.49** (0.43–0.58)	0.88 ^ns^ (0.80–0.94)	0.75* (0.69–0.84)	0.008
Serum triglycerides (mmol/L)	0.50 (0.49–0.64)	0.89 ^ns^ (0.86–1.21)	0.37^*^ (0.32–0.43)	0.35^*^ (0.34–0.42)	0.64 ^ns^ (0.42–1.05)	0.30^**^ (0.27–0.32)	0.009
Serum HDL cholesterol (mmol/L)	0.67 (0.58–0.72)	0.12^###^ (0.11–0.12)	0.45^*^ (0.43–0.51)	0.46^*^ (0.40–0.52)	0.41^ns^ (0.37–0.46)	0.45^ns^ (0.45–0.50)	0.003
Serum VLDL cholesterol (mmol/L)	0.10 (0.10–0.13)	0.18^ns^ (0.17–0.24)	0.07* (0.06–0.09)	0.07* (0.07–0.08)	0.13^ns^ (0.08–0.21)	0.06** (0.05–0.06)	0.009
Serum LDL cholesterol (mmol/L)	0.35 (0.34–0.36)	1.02^#^ (0.85–1.03)	0.35^*^ (0.33–0.36)	0.10^***^ (0.09–0.14)	0.41^ns^ (0.35–0.43)	0.26* (0.24–0.40)	0.001
Atherogenic index (LDL/HDL)	0.5 (0.5–0.6)	11.8^##^ (11.5–12.5)	0.9^ns^ (0.6–0.9)	0.1^***^ (0.1–0.1)	1.2^ns^ (0.8–1.5)	0.5^*^ (0.4–1.0)	<0.001
Coronary risk index (TC/HDL)	0.56 (0.51–0.59)	7.92^##^ (7.76–8.53)	0.73^ns^ (0.64–0.82)	0.23^***^ (0.22–0.28)	0.90^ns^ (0.66–1.28)	0.58* (0.49–0.97)	<0.001

Data represent median (Q1–Q3) of 5–6 rats; ^1^Kruskal–Wallis rank sum test; Significant difference from normal control: ^#^*P* < 0.05; ^##^*P* < 0.01; ^###^*P* < 0.001; Significant difference from CKD control: ^*^P < 0.05; ^**^P < 0.01; ^***^P < 0.001; No significant difference from CKD control: ns (*P* > 0.05). MTD: Minimal therapeutic dose; RD: Reduced dose; CKD: Chronic kidney disease; HDL: High-density lipoprotein; VLDL: Very low-density lipoprotein; LDL: Low-density lipoprotein.

### Kidney mass and histopathology

**Figure 5.**
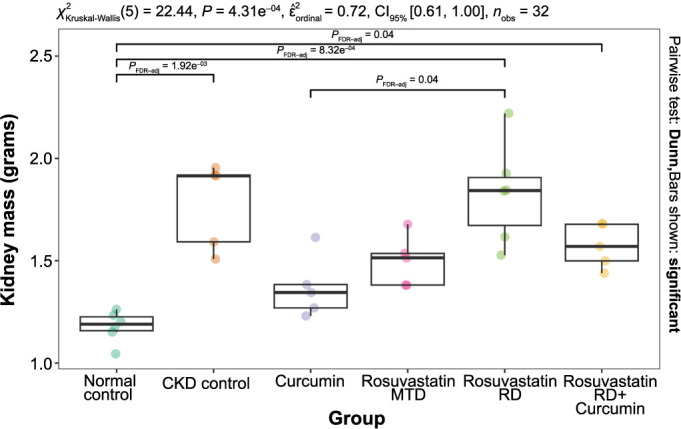
**The effects of curcumin, rosuvastatin in an MTD, rosuvastatin in an RD, and the combination of rosuvastatin RD and curcumin on kidney mass (g) in rats with adenine-induced CKD.** The normal control group was fed a standard diet, and the CKD control group was fed 0.75% w/w adenine in the standard diet. MTD: Minimal therapeutic dose; RD: Reduced dose; CKD: Chronic kidney disease.

The CKD control group showed a significantly higher kidney mass (1.92, IQR 1.59–1.92 vs 1.19, IQR 1.16–1.23; *P* ═ 0.002) compared to the normal control group. Lower kidney mass was observed in the curcumin group compared to other treatment groups, albeit still higher than in the control group (Figure 5).

The rats in the normal control group had normal renal architecture.

The renal histological structure in the CKD control group showed marked alterations in the cortical substance. In some cortical areas, focal hypertrophy of proximal nephrocytes was observed, resulting in a pseudostratified appearance of the epithelium. The proximal tubules were dilated, showing signs of apoptosis, degeneration, atrophy, and desquamation of nephrocytes. Some degenerated tubules showed signs of reorganization. Distal tubules showed similar changes in a milder form. Numerous brownish crystals were observed in the tubular system. The peritubular basement membranes were thickened. Altered tubules were surrounded by a thickened interstitium due to inflammatory cell infiltration and interstitial fibrosis. Renal corpuscles showed signs of early sclerosis, along with a thickened parietal layer of Bowman’s capsule, due to the thickening of the epithelium and associated basement membrane. In certain areas, signs of fibrosis could also be observed around the renal corpuscles.

Rats treated with curcumin alone or in combination with rosuvastatin RD displayed significantly more preserved renal histological structures compared to rats in the CKD control group. The signs of kidney damage, such as inflammation, interstitial fibrosis, and tubule degeneration and dilation, were notably milder in these groups.

**Figure 6.**
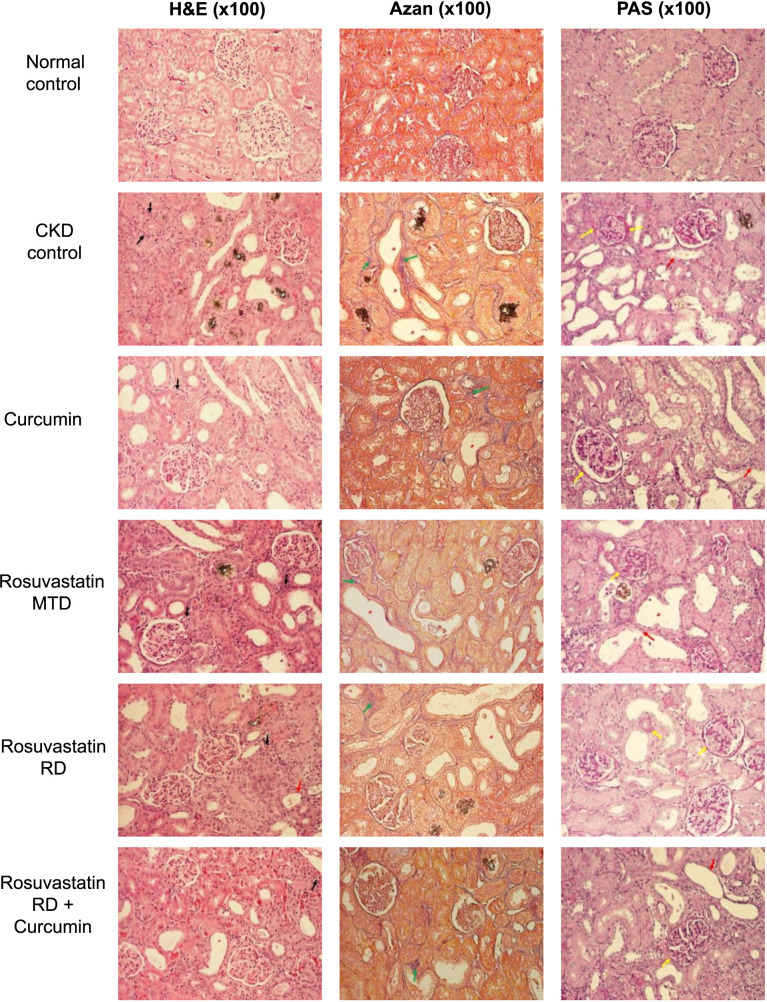
**Histopathological renal changes (light microscopy after sections stained with hematoxylin/eosin, Azan, and PAS) in rats with adenine-induced CKD treated with curcumin, rosuvastatin in an MTD, rosuvastatin in an RD, and the combination of rosuvastatin RD and curcumin.** The normal control group was fed a standard diet, and the CKD control group was fed 0.75% w/w adenine in the standard diet (black arrow—inflammation; green arrow—fibrosis; red arrow—tubular degeneration; yellow arrow—thickening of the basement membrane; white asterisk—crystal; and red asterisk—tubular dilation). PAS: Periodic acid-Schiff; MTD: Minimal therapeutic dose; RD: Reduced dose; CKD: Chronic kidney disease.

Rats treated with rosuvastatin MTD or rosuvastatin RD displayed marked alterations in the cortical substance with signs of damaged renal structure, such as inflammation, interstitial fibrosis, and tubule degeneration and dilation, similar to changes observed in the CKD control group (Figure 6 and Table 3).

**Table 3:** Semiquantitative analysis of the kidney in rats with adenine-induced CKD treated with curcumin, rosuvastatin in an MTD, rosuvastatin in an RD, and the combination of rosuvastatin RD and curcumin. The normal control group was fed a standard diet, and the CKD control group was fed 0.75% w/w adenine in the standard diet

**Treatment group**						
**Variable**	**Normal control (*N* ═ 6)**	**CKD control (*N* ═ 5)**	**Curcumin (*N* ═ 5)**	**Rosuvastatin MTD (*N* ═ 5)**	**Rosuvastatin RD (*N* ═ 6)**	**Rosuvastatin RD + Curcumin (*N* ═ 5)**
Inflammation	0	3+/4+	2+	3+	3+	2+
Interstitial fibrosis	0	3+	2+	3+	3+	2+/3+
Tubular degeneration	0	3+	2+	3+	2+/3+	2+
Tubular dilation	0	3+	2+	3+	3+/4+	2+
Crystals	0	3+	1+	3+	2+	1+/2+

0 (no changes), 1+ (minimal changes), 2+ (mild changes), 3+ (moderate changes), and 4+ (severe changes). MTD: Minimal therapeutic dose; RD: Reduced dose; CKD: Chronic kidney disease.

## Discussion

This is the first study to investigate the use of a combination of curcumin and rosuvastatin in CKD and associated dyslipidemia. While rosuvastatin in an MTD (5 mg/day) showed a beneficial effect only on the lipid profile and serum albumin levels, both curcumin alone and its combination with an RD of rosuvastatin improved both renal function and morphology, as well as lipid status, in adenine-induced CKD in rats. In contrast, an RD of rosuvastatin given alone showed no treatment potential, and both rosuvastatin doses impaired renal morphology.

Our main finding of the ability of curcumin alone, as well as its combination with rosuvastatin RD, to reverse renal functional and structural alterations in rats treated with 0.75% w/w adenine confirmed curcumin’s protective effect on kidney function and structure, as reported in previous studies [13, 14]. Besides an earlier pharmacokinetic study [21] that showed that orally administered curcumin with rosuvastatin significantly increased rosuvastatin concentration in rat and dog plasma, their pharmacodynamic interaction was also suggested. Namely, several studies have demonstrated that curcumin and rosuvastatin act through similar targets and molecular mechanisms, indicating the potential for their synergistic use as a therapeutic option for various diseases, including dyslipidemia [27–30]. In our study, both treatments with curcumin alone and in combination with rosuvastatin RD resulted in better renal function, with lower serum creatinine levels and milder renal morphological alterations, including inflammation, interstitial fibrosis, and tubular degeneration, as well as better lipid status, with lower levels of serum triglycerides, serum VLDL, and LDL cholesterol compared with levels in CKD control rats. Treatment with curcumin alone also resulted in significantly higher eGFR, lower serum uric acid levels, and higher serum HDL cholesterol, while the combined therapy provided additional positive effects on serum albumin levels, as well as cardiovascular protective effects with lower total serum cholesterol and lower both AI and CRI compared to untreated rats with CKD.

As reported in previous studies [13, 22, 31, 32], feeding rats a 0.75% w/w adenine-supplemented diet for 24 days is accepted as a reliable animal model of CKD that mimics changes occurring in humans but without a high mortality rate [33]. Compared to the 5/6 nephrectomy model, the use of adenine in the induction of CKD has several advantages, including avoidance of surgical manipulation and the development of different degrees of kidney disease, from mild to moderate and severe, depending on the dose and duration of adenine administration [34]. In this study, rats with CKD had significantly lower levels of eGFR and significantly higher levels of BUN and serum creatinine, indicating a decline in kidney function. Furthermore, albuminuria, along with interstitial fibrosis, early sclerosis of renal corpuscles, damaged nephrocytes, and a large amount of brown crystals in the lumina of proximal tubules of rats with CKD indicated severe kidney damage. However, in our study, four lethal outcomes were recorded among rats with CKD, and those rats also exhibited significant body mass loss (31.25%). Similar findings have been seen in previous studies [35–37]. Compared to the rats with CKD, in rats treated with curcumin and its combination with rosuvastatin RD body mass was not significantly different compared to the body mass of normal control rats on the last day of the experiment. The possible explanation may be the curcumin’s ability to reduce muscle atrophy and retain muscle function by increasing the cross-sectional area of myofibers [38]. Additionally, curcumin significantly reduced serum uric acid levels and consequently attenuated muscle atrophy and body mass loss in rats with CKD.

Curcumin’s renoprotective effects could be explained by its antioxidant and anti-inflammatory properties due to its chemical structure of hydroxyl groups in the phenolic rings, which regulate inflammatory signaling pathways, inhibit the production of inflammatory mediators, and reduce ROS [39–41]. Its antioxidant capacity was also proven in our previous *in vitro* study, where, compared to ascorbic acid, curcumin showed a similar radical scavenging capacity (RSC). Furthermore, its RSC was concentration-dependent, as the percentage of RSC increased with the increase in curcumin concentrations. This ability of curcumin could be attributed to the CH_2_ group and phenolic group of the ß-diketone moiety [42].

Hypoalbuminemia and albuminuria correlate with a decline in eGFR and are strong predictors of unfavorable disease outcomes, including mortality due to severe glomerulopathy [43]. Previous findings on the effects of curcumin on albuminuria vary; one study suggested no effects in doxorubicin-induced renal injury after eight weeks of curcumin treatment [18], while another study showed that curcumin significantly reduced albuminuria in a rat model of diabetic nephropathy [44]. Furthermore, in the other study of adenine-induced CKD, chronic supplementation with three different doses of curcumin (50, 100, and 150 mg/kg) reduced proteinuria compared to the CKD control group [14]. In our study, rats with CKD exhibited 3.1 times higher value of albuminuria compared to the normal control rats, but no treatment affected albuminuria significantly. In fact, both curcumin alone and its combination with rosuvastatin RD decreased albuminuria by 2.4 and 2.3 times, respectively, compared to untreated rats with CKD, but failed to achieve statistical significance. A possible explanation for our result may be the fact that adenine caused tubular atrophy and increased glomerular capillary permeability, leading to massive albuminuria, for which 24 days of treatment with curcumin were not sufficient to reverse.

Oxidative stress and inflammation are mechanisms involved in CKD-induced dyslipidemia, which often affects CKD progression [45]. The combined therapy with curcumin and rosuvastatin RD also improved the lipid status and corrected AI and CRI in rats with CKD, resulting in more beneficial effects on the lipid profile compared to curcumin alone. The possible explanation for this result may be attributed to the antioxidant and anti-inflammatory activity the combination demonstrated in our previous *in vitro* study [46]. Curcumin’s antilipemic effect observed in our study was mainly consistent with the results of previous studies on CKD and associated dyslipidemia in animal models [14, 15, 47, 48]. Previous studies reported that curcumin reduces the level of triglycerides by increasing the activity of lipoprotein lipase (LPL) [49, 50]. Curcumin increases serum HDL levels probably due to its ability to modulate markers of HDL function, as shown in a previous study where curcumin increased lecithin-cholesteryl acyltransferase and Apolipoprotein AI (Apo-AI) in rats and hamsters fed a high-fat diet [51, 52], as well as lowered induced activity of cholesteryl ester transfer protein in the liver and plasma of rabbits fed a high-cholesterol diet [53]. The proposed mechanism by which curcumin decreases levels of LDL cholesterol is by stimulating LDL receptors, as shown in human hepatoma-derived HepG2 cells [54] or mouse macrophages [55].

Contrary to previous reports of cholesterol-lowering effects of curcumin in animals and humans [44, 56, 57], this effect was seen in our study only in groups treated with rosuvastatin MTD and the combined therapy. A similar finding was reported in a human study where curcumin alone was also not able to significantly reduce serum total cholesterol levels after six months of treatment [58].

Consistent with the results of previous studies [59, 60], treatment with rosuvastatin MTD resulted in significantly better overall lipid status and both AI and CRI compared with untreated rats with CKD in our study. However, as mentioned earlier, while several studies reported its beneficial effects on renal function [61, 62], other studies reported that rosuvastatin causes renal tissue injury [7, 8] and could lead to hematuria and proteinuria, especially in patients with CKD [6]. This aligns with our findings, as treatment with both rosuvastatin doses impaired renal morphology, and of all markers of renal function, it positively affected only serum albumin levels, as also seen with the combined rosuvastatin RD and curcumin treatment.

Our study had several limitations. First, four lethal outcomes were recorded among rats with CKD before the end of the experiment, affecting the statistical power to detect small differences between the groups. Second, we designed the study based on literature data, but 24 days may not be long enough to capture the long-term effects of the investigated treatments. Third, the observed benefits of the studied combination were explained using only one *in vitro* study. Although the rationale behind the use of the reduced rosuvastatin dose was its expected pharmacokinetic and pharmacodynamic interactions with curcumin, as well as its nephrotoxicity potential, the lack of measurement of rosuvastatin serum concentrations was an additional limitation of this study.

The results of this study may be of clinical relevance as they provide first-time evidence for a potential new therapy option for CKD and associated dyslipidemia. However, for better insight into the therapeutic potential of the combination of curcumin and an RD of rosuvastatin, larger animal trials are needed. Also, to ensure its clinical relevance and effectiveness in treating CKD and associated dyslipidemia, and to establish therapeutic protocols, rigorous clinical trials are required.

## Conclusion

The results of this study confirmed the beneficial effects of curcumin and provided new evidence for the beneficial renal and antilipemic effects of its combination with an RD of rosuvastatin in rats with adenine-induced CKD and associated dyslipidemia. In contrast to its combination with curcumin, rosuvastatin at a therapeutic dose showed a beneficial effect only on lipid status and serum albumin levels, while impairing renal morphology.

## Data Availability

The datasets generated or analyzed during the current study are available from the corresponding author upon reasonable request.
